# HER2 expression and genOmic characterization of rESected brain metastases from colorectal cancer: the HEROES study

**DOI:** 10.1038/s41416-023-02569-4

**Published:** 2024-02-12

**Authors:** Alessandra Anna Prete, Valentina Angerilli, Francesca Bergamo, Valentina Vettore, Chiara De Toni, Rossana Intini, Krisida Cerma, Gianmarco Ricagno, Riccardo Cerantola, Eleonora Perissinotto, Antonio De Rosa, Carlotta Ceccon, Jessica Gasparello, Luca Denaro, Alberto D’Amico, Franco Chioffi, Elena Carcea, Matteo Fassan, Sara Lonardi

**Affiliations:** 1 Medical Oncology 1, Veneto Institute of Oncology IOV–IRCCS, Padua, Italy; 2 Department of Medicine (DIMED), Surgical Pathology & Cytopathology Unit, University of Padua, Padua, Italy; 3 Academic Neurosurgery, Department of Neurosciences, University of Padua, Padua, Italy; 4 Division of Neurosurgery, Azienda Ospedaliera Università di Padova, Padua, Italy; 5 Veneto Institute of Oncology IOV–IRCCS, Padua, Italy; 6 Medical Oncology 3, Veneto Institute of Oncology IOV–IRCCS, Padua, Italy

**Keywords:** Prognostic markers, Tumour biomarkers

## Abstract

**Background:**

Little is known about prognostic factors of brain metastases (BM) from colorectal cancer (CRC). *HER2* amplification/overexpression (HER2+) was previously described; its impact on prognosis remains uncertain.

**Methods:**

In the translational study HEROES, extensive molecular analysis was performed on primary CRC (prCRC) and their matched resected BM by means of NGS comprehensive genomic profiling and HER2 status as assessed by immunohistochemical/ in situ hybridization. Count of tumour-infiltrating lymphocytes (TILs) was also performed. Primary objective: to describe the molecular landscape of paired BM/prCRC. Secondary objectives: to search for new prognostic biomarkers of outcome after BM resection: intracranial-only Progression-Free Survival (BM-iPFS), Progression-Free Survival (BM-PFS), and Overall Survival (BM-OS).

**Results:**

Out of 22 patients having paired samples of prCRC and BM, HER2+ was found on 4 (18%) BM, 3 (75%) of which also HER2+ in matched prCRC. Lower tumour mutation burden (HR 3.08; 95%CI 1.06–8.93; *p* = 0.0386) and HER2-negative BM (HER2neg) (HR 7.75;95%CI 1.97–30.40; *p* = 0.0033) were associated with longer BM-iPFS; HER2neg BM (HR 3.44; 95%CI 1.03–11.53; *p* = 0.0449) and *KRAS*^mut^ BM (HR 0.31; 95%CI 0.12–0.80; *p* = 0.0153) conferred longer BM-PFS. Longer BM-OS was found in pts with TILs-enriched (≥1.6/HPF) BM (HR 0.11; 95%CI0.01–0.91; *p* = 0.0403).

**Conclusions:**

This study shows HER2+ enrichment in both BM and their prCRC. TILs-enriched BM conferred better BM-OS.

## Introduction

Brain metastases (BM) represent a rare event in colorectal cancer (CRC), but carry an extremely poor prognosis. Their incidence is estimated to be around 1 to 3% [1], with a trend to increase as a consequence of the prolonged CRC overall survival. The rarity of BM from CRC, together with the technical difficulties to access BM tissue, limits the feasibility of prospective studies and molecular characterization, resulting in scarce information about their development and prevention.

Clinical factors with prognostic value in patients with BM from CRC have been previously studied: a dedicated nomogram created by Pietrantonio et al., shows how age, Karnofsky performance status, site and number of BM can impact survival [2]. On the other hand, the advances in molecular diagnostics improved insight into molecular pathways and alterations underlying BM development. In analogy with breast cancer and other malignancies, enrichment in *ERRB2* amplification/HER2 overexpression (HER2 + ) has been recently described in a cohort of patients with BM from gastrointestinal tumours [3]. HER2+ is rare in CRC, being detected in overall 1-2% of patients with CRC and no more than 5% of patients with *RAS/BRAF* wild-type disease [4–6]. In HER2-positive breast cancer, understanding tumour biology has been crucial to identify those patients at higher risk of BM development and therefore worthy of special surveillance, such as central nervous system periodic scan, for early diagnosis and treatment.

Another factor emerging from literature is the increased tendency to BM in CRC with *BRAF*^V600E^ mutation [7]: the aggressiveness of this CRC subtype is well known, but conclusive data about BM can hardly be drawn due to the rarity of both *BRAF*-mutated metastatic CRC (mCRC) (8–12% of the total mCRC) [8] and BM.

Recently, deficiency in homologous recombination (HRD) and mismatch repair (MMRD) were also described in tissue from BM compared to matched primary CRC tumours in a cohort of 19 patients [9]: this finding opens interesting scenarios in the genomic profiling of BM from CRC.

Moving from such a background, we designed the present study in order to describe genomic landscape and clinical characteristics of patients with BM from CRC, with a special focus on HER2 expression.

## Patients and methods

HEROES was a retrospective-prospective, translational study in which patients with resected BM from CRC and treated at our institution were enrolled to perform extensive molecular analysis of matched tissue from primary tumour and BM.

Criteria of inclusion were: i) histologically proven diagnosis of CRC, ii) diagnosis of BM, iii) availability of matched primary CRC tumour and BM tissue specimens. All the patients signed written informed consent; the protocol received approval from local Ethic Committee of Veneto Institute of Oncology IOV – IRCCS, and was conducted in accordance with the Declaration of Helsinki.

A dedicated database was created, in which both clinical and molecular characteristics were collected. Molecular characterization and tumour mutation burden (TMB) quantification were obtained by means of Next Generation Sequencing (NGS, FoundationOne CDx®). Furthermore, *RAS/BRAF* and Microsatellite Instability (MSI) were respectively assessed with MassArray (Myriapod Colon Status® kit) and immunohistochemistry (IHC) to validate NGS results. HER2 was assessed with IHC/in situ hybridization (ISH) using criteria previously reported in literature [10], and HER2 3+ overexpression or HER2 2+ overexpression with ISH-confirmed amplification were considered as HER2 HER2+. Tumour-infiltrating lymphocytes (TILs) were counted on hematoxylin-eosin-stained tissue sections; no immunohistochemical characterization was provided, so a quantitative-only assessment was conducted.

All the analyses were performed on matched primary tumour and BM tissue for each patient.

Primary objective of the study was to report the molecular landscape of paired tissues from primitive tumour and brain metastases, with special focus on HER2.

Secondary objectives were to search for new prognostic biomarkers in patients with resected brain metastases from CRC. Three survival endpoints were defined, all of them starting from BM resection: the first to the time of intracranial-only disease progression, death (any cause) or last follow-up, whichever occurred first (intracranial-only progression-free survival, BM-iPFS), the second to the disease progression in any site, death (any cause) or last follow up, whichever occurred first (progression free survival, BM-PFS), and the third to the time of death for any cause or at last follow up, whichever occurred first(overall survival, BM-OS). Canonical overall survival (OS), defined as the time from diagnosis of metastatic disease to death for any cause or last follow up, whichever occurred first., was also described.

### Statistical design

Given the exploratory, descriptive nature of this study, no formal statistical hypothesis was generated. The median follow-up was calculated using the reverse Kaplan-Meier method, starting from BM resection. Fisher’s exact test was applied to evaluate the distribution of clinical and molecular characteristics. Survival endpoints (BM-iPFS, BM-PFS, BM-OS, OS) were described using the Kaplan-Meier method, and Cox proportional hazards regression model was applied to calculate the hazard ratios (HR); given the low sample size, no multivariate analysis nor interactions tests were feasible. All tests were two sided.

Cut-offs for TMB and TILs were set with ROC curves. All the statistical analyses were performed using R software v.4.2.3.

## Results

### Clinical characteristics of study population

Out of 101 pts with BM from CRC treated at our Institution from 1 January 2010 until 31 December 2021, 22 (10 males/12 females) underwent BM resection and were thus included in the analysis. Of them, 11 (50%) were retrospectively enrolled while the remaining 11 (50%) patients were prospectively enrolled starting from 1st March 2018. The large majority of the overall population was composed by patients aged ≥70 (18 out of 22, 82%), with left colon or rectal cancer (19 out of 22, 86%); furthermore, 17 (77%) of the patients enrolled had also lung metastases, while liver metastases were found in 11 (50%) patients. BM were metachronous (onset > 6 months after prCRC diagnosis) in most cases (68 vs 32%); at the time of brain surgery, the majority of patients had both intra and extracranial disease (64 vs 36%); just three out of 22 patients had more than a single BM at the time of surgery (Table 1). Of the 22 patients included in the analysis, 6 (27%) did not receive any systemic antitumoral treatment for mCRC, while 7 (32%) received three or more lines of treatment. Two patients received immunotherapy in experimental trials, one of them bearing MSI-high (MSI-H) mCRC; importantly, none of them received anti-HER2 treatments (Supplementary Table 1).

**Table 1:** Clinical characteristics of study population.

	Patients *N* = 22 (%)
Age at first tumour diagnosis, years	
Median (IQR)	51 (47–65)
<70	4 (18)
≥70	18 (82)
Sex	
Male	10 (45)
Female	12 (55)
ECOG PS at baseline	
0	13 (59)
≥1	9 (41)
Stage at diagnosis	
I-II-II	11 (50)
IV	11 (50)
Synchronous vs metachronous metastases	
Sync (<6 months)	11 (50)
Meta (≥6 months)	11 (50)
Primary tumour location	
Right	3 (14)
Left	9 (41)
Extraperitoneal rectum	10 (45)
Primary tumour resection	
Yes	17 (77)
No	5 (23)
Liver metastases at any time	
Yes	11 (50)
No	11 (50)
Lung metastases at any time	
Yes	17 (77)
No	5 (23)
Lines of treatments received in total	
≤3	15 (68)
>3	7 (32)
Age at brain metastases resection, years	
Median (IQR)	58 (48–68)
<70	17 (77)
≥70	5 (23)
ECOG PS at the time of brain metastases resection	
0	8 (36)
≥1	14 (64)
Brain metastases presentation	
Synchronous	7 (32)
Metachronous	15 (68)
Tumour burden at the time of brain surgery	
Intracranial-only disease	8 (36)
Intra and extracranial disease	14 (64)
Number of brain metastases at the time of brain surgery	
1	19 (86)
>1	3 (14)

### Molecular and immunohistochemical landscape of matched CRC and BM

Molecular and immunohistochemical characteristics of BM were consistent with data reported in Literature: out of 22 analysed BM, HER2+ was documented in four (18%); three (14%) carried a *BRAF*^V600E^ mutation; two (9%) displayed MSI-H: therefore, HER2, *BRAF*^V600E^ and MSI-H enrichment in BM from CRC was confirmed.

Some heterogeneity between prCRC and corresponding BM was recognizable: out of four patients with HER2 + BM, only two displayed HER2+ also on matched prCRC; in the other 2 cases, HER2+ was acquired on BM, being not documented on corresponding prCRC. On the other hand, in one case HER2+ was lost from prCRC to coupled BM (Table 2). Therefore, in total 3 (14%) patients out of 22 had discordant *HER2* status between BM and matched prCRC (*p* = 1.000).

**Table 2:** Molecular and histological characteristics of matched BM and primitive CRC tissue.

	Primary tumour *N* = 22 (%)	Brain metastases *N* = 22 (%)	*p*-value
HER2			
Ampl	3 (14)	4 (18)	1.000
Non ampl	19 (86)	18 (82)	
Not evaluable	*0*	*0*	
KRAS			
WT	5 (24)	8 (36)	0.310
Mut	16 (76)	14 (64)	
Not evaluable	1	*0*	
NRAS			
WT	19 (95)	21 (95)	1.000
Mut	1 (5)	1 (5)	
Not evaluable	2	*0*	
BRAF			
WT	19 (95)	19 (86)	0.608
Mut	1 (5)	3 (14)	
Not evaluable	2	*0*	
MSI			
MSS	20 (91)	20 (91)	1.000
MSI-H	2 (9)	2 (9)	
Not evaluable	0	*0*	
TMB			
High ≥ 5	7 (44)	10 (48)	1.000
Low < 5	9 (56)	11 (52)	
Not evaluable	6	*1*	
TILs			
High ≥ 1.6	8 (44)	5 (25)	0.307
Low < 1.6	10 (56)	15 (75)	
Not evaluable	4	*2*	
Grading			
G1/G2	15 (71)	5 (71)	1.000
G3/G4	6 (29)	2 (29)	
Not evaluable	1	15	
TP53			
WT	4 (20)	5 (23)	1.000
Mut	16 (80)	17 (77)	
Not evaluable	2	0	
APC			
WT	4 (20)	4 (18)	1.000
Mut	16 (80)	18 (89)	
Not evaluable	2	0	
PIK3CA			
WT	15 (75)	20 (91)	0.229
Mut	5 (25)	2 (9)	
Not evaluable	2	0	

Looking at the three patients with *BRAF* mutations on BM, in one case *BRAF*^V600E^ mutation was documented on both prCRC and matched BM; in another patient, the mutation was detected on BM but not on the corresponding prCRC; for the third patient, *BRAF* on prCRC was not evaluable, so the comparison was not feasible.

*KRAS* mutations were more frequent than expected (16 out of 22, 76%) and were consistent between matched primary tumour and BM; only one patient out of 22 had a *NRAS* mutation (p. Q61R), which was found in both primary tumour and BM.

Other frequent mutations found by means of NGS in BM were observed in the *TP53* (17 out of 22 BM, 77%), *APC* (18 out of 22 BM, 89%) and *PIK3CA* (2 out of 22 brain metastases, 9%) genes. The distribution of *PIK3CA* mutations was heterogeneous between coupled samples, being gained from primary tumour to BM in one patient and lost in other four cases (Table 2).

Median TMB value was 4 mut/Mb both on prCRC specimens (range 1 to 115 mut/Mb) and on BM (range 1 to 57 mut/Mb). Using ROC curves as formerly described, TMB was defined as high if ≥ 5.02 mut/Mb. Median TILs number was 2/HPF (range 1 to 5/HPF) on primary samples and 0.6/HPF (range 0 to 6/HPF) on BM. TILs were defined as high if ≥ 1.6/HPF. With such cut-offs, respectively 10 (48%) and 5 (25%) BM specimens had high TMB and TILs, being both consistent between matched primary tissue and BM.

### Impact of clinical, molecular and immunohistochemical characteristics on survival

**Fig. 1:**
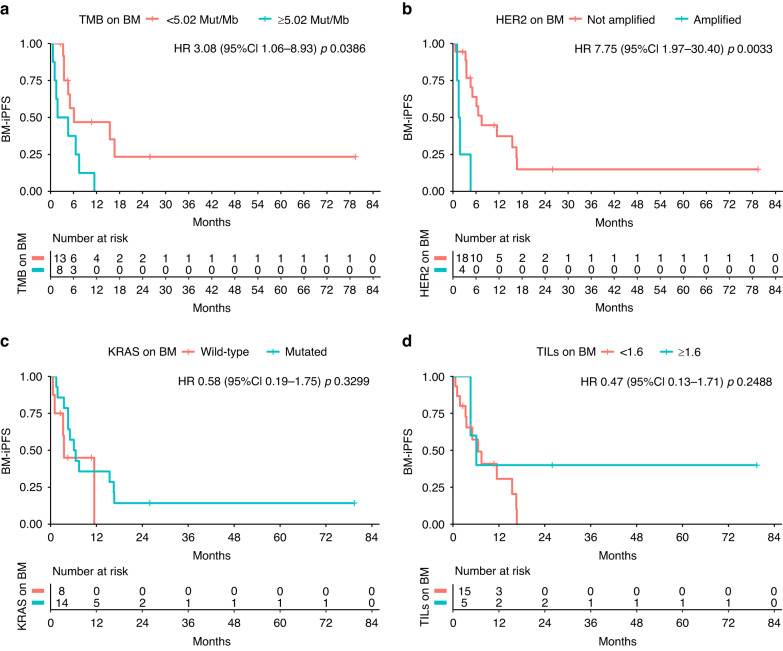
BM-iPFS. In our analyses, intracranial progression-free survival from BM (brain metastasis) resection (BM-iPFS) was improved in case of low TMB (Tumor Mutation Burden) (<5.02 Mut/Mb) (**a**) and absence of HER2 amplification (HER2+) (**b**) on BM; on the contrary, no correlation was observed with KRAS status (**c**) or TILs (Tumor Infiltrating Lymphocytes) on BM (**d**).

At a median follow-up of 45.89 months (95% CI 17.73 to 88.03), factors positively influencing BM-iPFS were low TMB (6.12 vs 3.23 months; HR 3.08; 95% CI 1.06 - 8.93; p value = 0.0386) and absence of HER2+ on brain metastases (7.47 vs 1.68 months; HR 7.75; 95% CI 1.97 −30.40; *p* value = 0.0033). On the contrary, *KRAS* mutations did not have a significant impact on BM-iPFS (HR 0.58; 95% CI 0.19–1.75; *p* value = 0.3299); as well, no differences in BM-iPFS were detected depending on TILs number on BM (HR 0.47; 95% CI 0.13–1.71; *p* value = 0.2488) (Fig. 1).

**Fig. 2:**
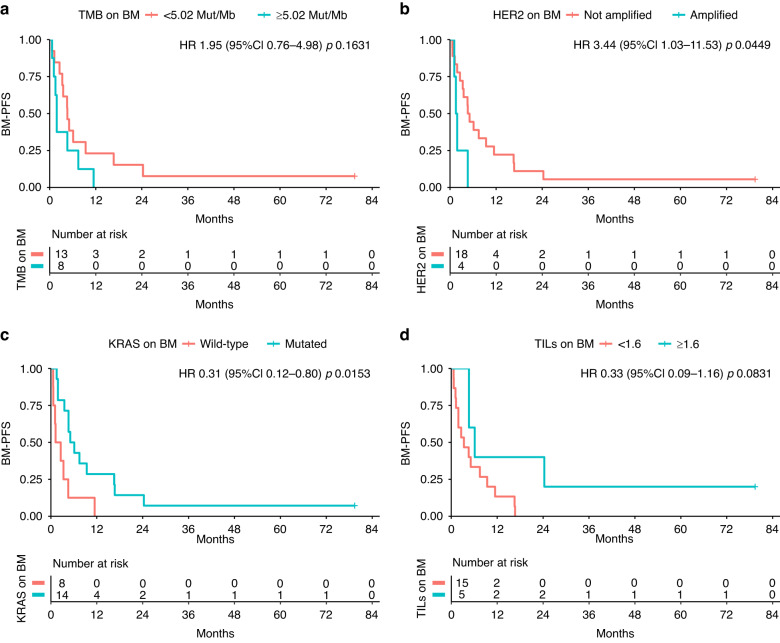
BM-PFS. No correlation was confirmed between any-site progression free survival from brain metastasis (BM) resection (BM-PFS) and TMB (Tumor Mutation Burden) on BM (**a**); on the other hand, HER2 amplification (HER2+) (**b**) and KRAS mutations (**c**) on BM yielded better BM-PFS. A non-statistically significant advantage in BM-PFS was observed in case of TILS ≥ 1.6 on BM (**d**).

The absence of *HER2*+ on BM had also a positive impact on BM-PFS (4.84 vs 1.68 months; HR 3.44; 95%CI 1.03–11.53; *p* = 0.0449), as well as *KRAS* mutations (1.90 vs 5.60 months; 0.31; 95% CI 0.12–0.80; *p* value = 0.0153). On the contrary, TMB and TILs did not have a clear impact on BM-PFS (respectively HR 1.95; 95% CI 0.76–4.98; *p* value = 0.1631 and HR 0.33; 95% CI 0.09–1.16; *p* value = 0.0831) (Fig. 2).

**Fig. 3:**
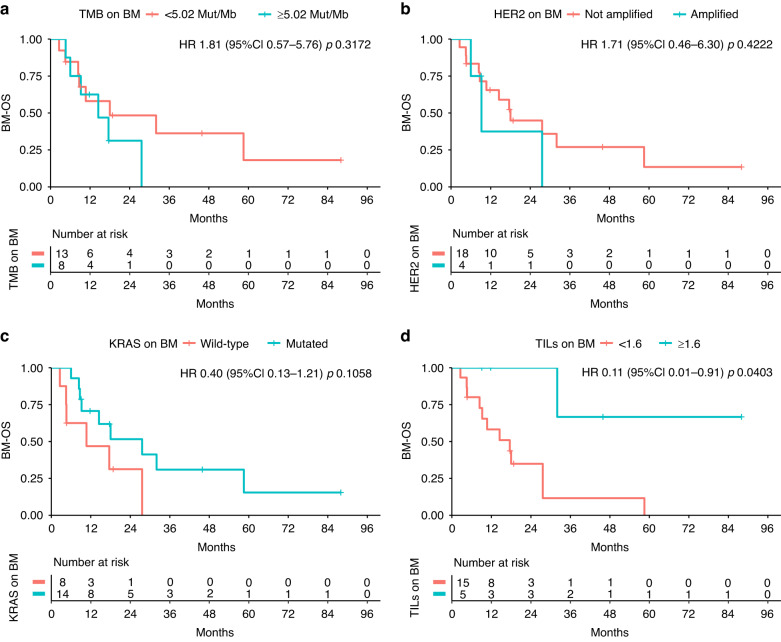
BM-OS. Looking at overall survival from brain metastasis (BM) resection (BM-OS), no correlation was demonstrated with TMB (Tumor Mutation Burden) (**a**), HER2 amplification (HER2+) (**b**) and KRAS mutations (**c**) on BM. Instead, statistically significant advantage was observed in case of TILS ≥ 1.6 on BM (**d**).

**Fig. 4:**
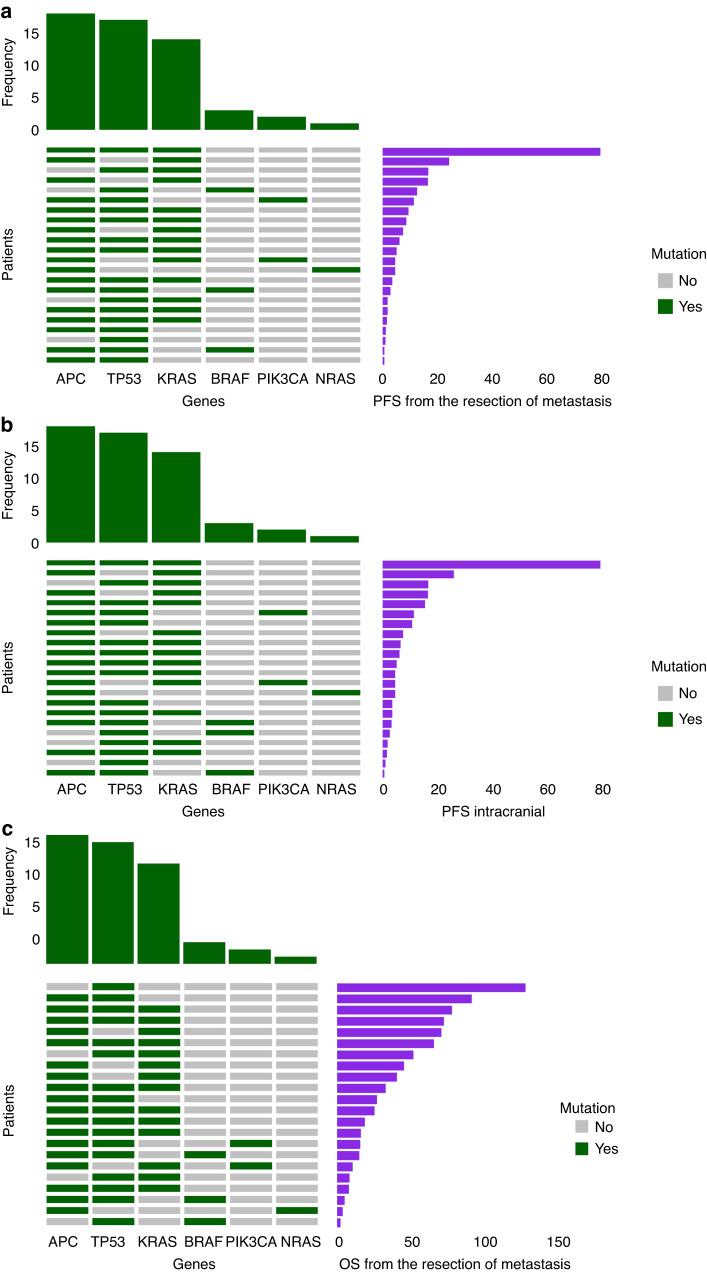
Impact of single genes. As depicted below, neither any-site (BM-PFS) (**a**) nor intracranial progression free survival (BM-iPFS) (**b**) and overall survival from brain metastasis (BM) resection (BM-OS) (**c**) were influenced by any of the molecular alterations detected with Next Generation Sequencing (NGS).

OS from the diagnosis of metastatic disease was 37.20 months (CI 95%: 21.30 - NA). Longer BM-OS was found in pts with higher TILs (≥1.6) on BM (*p* value = 0.0403), whereas TMB, HER2 status, and *KRAS* mutations had no clear effect (Fig. 3). As well, none of the other mutations individuated with NGS had an impact on survival (Fig. 4).

## Discussion

In this study, extensive molecular landscape of BM from CRC was described, providing unique data on this poorly explored field of study. In our experience, both BM-iPFS and BM-PFS were positively influenced by absence of HER2 + . TMB < 5.02 mut/Mb conferred better BM-iPFS also; however, it must be pointed out that this is not a recognised cut-off, but was set as described above in order to better characterize the relatively small sample size of the present study. The presence of *KRAS* mutations was related to better BM-PFS only.

Considering these results, it might be argued that this study suggests a prognostic value for HER2, TMB and *KRAS* in patients with BM from CRC. Nevertheless, caution should be used in interpreting and generalizing our results. Indeed, apart from the small sample size, our cohort is also strictly selected from a clinical point of view: patients diagnosed with BM are deemed amenable to surgical resection only in case of optimal clinical conditions, small and/or single brain lesions with favourable localization; furthermore, it is more likely that these patients are referred to high volume and expertise surgical centres. Accordingly, our case series was composed of 22 individuals selected out of 101 patients with BM from CRC, because they were the only ones who were declared eligible for surgery. Thus, it would be more appropriate to infer that our results might guide in clinical management of patients amenable to neurosurgery for BM from CRC, and not to all of them.

The effect of HER2 on prognosis is intriguing and deserves special discussion. An interaction test between HER2 and other known strong prognostic factors such as *BRAF* or ECOG PS could provide further insight on their relative weight on prognosis; unfortunately, the sample size was not sufficient to proceed with further analyses. Nonetheless, the negative influence on BM-iPFS and BM-PFS conferred by HER2+ documented in our work could be explained by three main factors: negative impact of HER2+ per se on prognosis, absence of targeted therapies against *HER2* at the time of treatment, and resistance conferred by HER2+ to anti-EGFR.

Resistance to anti-EGFR has been widely documented in patients with HER2 + CRC [11–13]; on the contrary, the prognostic role of *HER2*+ has not been well established in CRC; nevertheless, the tendency to worse outcomes in survival has been reported in patients with HER2 + CRC [14]. More recently, worse relapse-free survival (RFS) was described in patients with liver metastases from CRC displaying HER2 + . In our series, only three patients out of 22 (14%) received anti-EGFR and none of them received anti-HER2 therapy. According to the results of the HERACLES trial [6] and, more recently, to the results of the DESTINY-CRC01 trial [15], anti-HER2 agents could revert the negative impact on prognosis exerted by HER2 + . A special mention should be reserved to the intracranial activity of these drugs: in particular, trastuzumab is known to be unable to pass the blood-brain barrier. Of note, unexpectedly high rate of BM was observed in relationship with treatment-prolonged survival after trastuzumab and lapatinib in patients with *HER2*-amplified CRC [3]. Conversely, trastuzumab-deruxtecan showed remarkable intracranial activity in a dedicated phase II trial [16]; however, this study was conducted on patients with breast cancer, and no studies are currently available specifically addressing the activity of trastuzumab-deruxtecan on BM from CRC.

Putting our results into a purely clinical context, the lesson learnt from this experience was to always bear in mind the chance of BM development in patients with HER2 + CRC: thus, symptoms suggesting cerebral localizations should be carefully explored in this special population. Moreover, especially in case of long survivors, including cerebral imaging in routine follow-up might become reasonable if our data will be further confirmed.

Moving from the case of *HER2*, isolating specific molecular alterations potentially predictive of BM development could be intriguing: in this work, together with *HER2*, also *KRAS*, *BRAF*^V600E^, *TP53* and *APC* mutations were observed in BM. On the other hand, we described how some of these alterations could be either lost or gained from prCRC to BM, suggesting an evolving molecular landscape from primary tumour to BM. Previous studies described intratumor heterogeneity of HER2 expression between prCRC and BM, documenting discordant HER2 status between primary tumour and matched metastases, both intra and extracranial: in these studies, the impact of HER2 discordance on prognosis is unclear [17, 18]; on the other hand, the indirect weight of the heterogeneity between prCRC and metastases on prognosis is intuitive, because molecular modifications could lead to increased therapeutic chances.

Safe access to brain tissue for molecular characterization would be useful to drive clinical choices, especially in the era of target therapy; given the well-known limits related to intracranial surgical procedures, liquid biopsy could be of special interest in these situations. Unfortunately, scarcity of circulating tumour DNA (ctDNA) has been documented in plasma in case of BM from solid tumours, as well as in case of central nervous system primary tumours [19]. Notwithstanding, cerebrospinal fluid (CSF) is emerging as a source of ctDNA from brain lesions: in fact, several actionable mutations have been identified in CSF-ctDNA, and there is some suggestion that CSF-ctDNA is more accurate than blood ctDNA in reproducing private molecular alterations of BM from breast cancer, being also detectable only in patients with BM, and decreasing accordingly to response to systemic treatments and/or intracranial surgery [20]. No specific data are available regarding CRC; however, these previous experiences could represent a valid starting point for further development.

A finding that is worth of special mention regards TMB: in our report, TMB < 5.02 mut/Mb was related to better BM-iPFS. This result must be interpreted with caution: high TMB has been related to better prognosis in both limited-stage CRC [21] and metastatic setting [22]; furthermore, predictive value of high TMB in case of therapy with immune checkpoint inhibitors has been demonstrated [23]. However, in our series only two patients received immune checkpoint inhibitors. Although FDA approval of pembrolizumab for patients with solid tumors with high TMB took as cutoff 10 mut/Mb, this threshold is still object of debate since it could be dependent on histology [24, 25] and on the assay employed to determine the TMB value itself. For example, in the study from Innocenti et al., positive prognostic value was attributed to high TMB in patients with metastatic MSS CRC; in this study, the threshold to define high TMB was 8 mut/Mb. Of note, in our cohort, the large majority of patients had left-sided CRC: therefore, a tendency to lower TMB was predictable.

## Conclusion

Even with the limitation of small sample size, the HEROES study supports HER2+ enrichment in both prCRC and BM from CRC. Absence of *HER2*+ seems to confer better BM-iPFS and BM PFS in patients with resected BM from CRC. In the future, larger studies and new techniques like liquid biopsy could be important to better assess the molecular landscape evolution of BM from CRC in order to individuate new prognostic and predictive factors.

### Supplementary information

Supplemental material

REMARK checklist

## Data Availability

The data generated in this study are available within the article.
