# Supplementary material for: HER2 expression and genOmic characterization of rESected brain metastases from colorectal cancer: the HEROES study
Source: Br J Cancer. 2024 Feb 12;130(8):1316–23. doi: 10.1038/s41416-023-02569-4 (PMC11014920; doi:10.1038/s41416-023-02569-4)
Supplement: Supplementary file 1 — Supplemental material [file 41416_2023_2569_MOESM1_ESM.docx]

**Supplementary Material**

**Index**

- Supplementary Table 1: page 2

|  | **Patients**  **N=22**  **(%)** |
| --- | --- |
| **Lines of treatment received** |  |
| 1 | 8 (36) |
| 2 | 1 (5) |
| ≥ 3 | 7 (32) |
| No chemotherapy for metastatic disease | 6 (27) |
| **1^st^ line treatment** |  |
| Chemo doublet + anti VEGF | 8 (50) |
| Chemo doublet + anti EGFR | 0 |
| Chemo triplet +/- anti VEGF | 3 (19) |
| Immunotherapy | 1 (6) |
| Other* | 4 (25) |
| *No treatment* | *6* |
| **2^nd^ line treatment** |  |
| Chemo + anti VEGF | 4 (50) |
| Chemo + anti EGFR | 1 (12,5) |
| Immunotherapy | 1 (12,5) |
| Other* | 2 (25) |
| *No treatment* | *14* |

*Folfox, Folfiri, Folfoxiri Bevacizumab Nivolumab in trial, capecitabine bevacizumab

**Supplementary Table 1.** Details on antitumoral systemic treatments administered
